# Toward a nomenclature consensus for diverse intelligent systems: Call for collaboration

**DOI:** 10.1016/j.xinn.2024.100658

**Published:** 2024-06-17

**Authors:** Brett J. Kagan, Michael Mahlis, Anjali Bhat, Josh Bongard, Victor M. Cole, Phillip Corlett, Christopher Gyngell, Thomas Hartung, Bianca Jupp, Michael Levin, Tamra Lysaght, Nicholas Opie, Adeel Razi, Lena Smirnova, Ian Tennant, Peter Thestrup Wade, Ge Wang

**Affiliations:** 1 Cortical Labs, Brunswick, VIC 3056, Australia; 2 Wellcome Centre for Human Neuroimaging, University College London, London WC1N 3AR, UK; 3 University of Vermont, Burlington, VT 05405, USA; 4 Centre for Professional Communication, Singapore Institute of Technology, Singapore 138683, Singapore; 5 Department of Psychiatry, Yale University, New Haven , CT 06511, USA; 6 Murdoch Children’s Research Institute, Parkville, VIC 3052, Australia; 7 Centre for Alternatives to Animal Testing (CAAT), Bloomberg School of Public Health and Whiting School of Engineering, Johns Hopkins University, Doerenkamp-Zbinden Chair for Evidence-based Toxicology, Baltimore , MD 21205, USA; 8 CAAT-Europe, University of Kostanz, 78464 Kostanz, Germany; 9 Department of Neuroscience, Central Clinical School, Monash University, Prahran, VIC 3004, Australia; 10 Allen Discovery Centre at Tufts University, Medford , MA 02155, USA; 11 Yong Loo Lin School of Medicine, National University of Singapore, Singapore 117597, Singapore; 12 Faculty of Medicine and Health Sydney School of Public Health, The University of Sydney, Camperdown, NSW 2050, Australia; 13 Department of Medicine, University of Melbourne, Parkville, VIC 3010, Australia; 14 Turner Institute for Brain and Mental Health, Monash University, Clayton, VIC 3168, Australia; 15 Wellcome Centre for Human Neuroimaging, University College London, London WC1N 3AR, UK; 16 Center for Alternatives to Animal Testing (CAAT), Bloomberg School of Public Health and Whiting School of Engineering, Johns Hopkins University, Baltimore , MD 21205, USA; 17 Faculty of Engineering, AgriTech and Environment, Anglia Ruskin University, Peterborough PE1 5BW, UK; 18 Interacting Minds Centre, Aarhus University, 8000 Aarhus C, Denmark; 19 Wellcome Centre for Human Neuroimaging, University College London, London WC1N 3AR, UK; 20 Department of Biomedical Engineering, School of Engineering, Biomedical Imaging Center, Center for Biotechnology and Interdisciplinary Studies, Rensselaer Polytechnic Institute, Troy, NY 12180, USA; 21 Department of Biochemistry and Pharmacology, University of Melbourne, Parkville, VIC 3010, Australia

## Abstract

Disagreements about language use are common both between and within fields. Where interests require multidisciplinary collaboration or the field of research has the potential to impact society at large, it becomes critical to minimize these disagreements where possible. The development of diverse intelligent systems, regardless of the substrate (e.g., silicon vs. biology), is a case where both conditions are met. Significant advancements have occurred in the development of technology progressing toward these diverse intelligence systems. Whether progress is silicon based, such as the use of large language models, or through synthetic biology methods, such as the development of organoids, a clear need for a community-based approach to seeking consensus on nomenclature is now vital. Here, we welcome collaboration from the wider scientific community, proposing a pathway forward to achieving this intention, highlighting key terms and fields of relevance, and suggesting potential consensus-making methods to be applied.

## Background

The use of language to describe specific phenomena for scientific and public discourse is complex and can be highly contentious for emerging science and technology. This is because human language is value laden, and terminology can have normative implications, especially when uncertainty surrounds the ontological status and moral concern for the scientific objects or artifacts of study. Nevertheless, effective scientific communication requires given signifiers (the sign for a given concept) to be understood in a specific context and ideally for a distinct concept.

**Figure 1:**
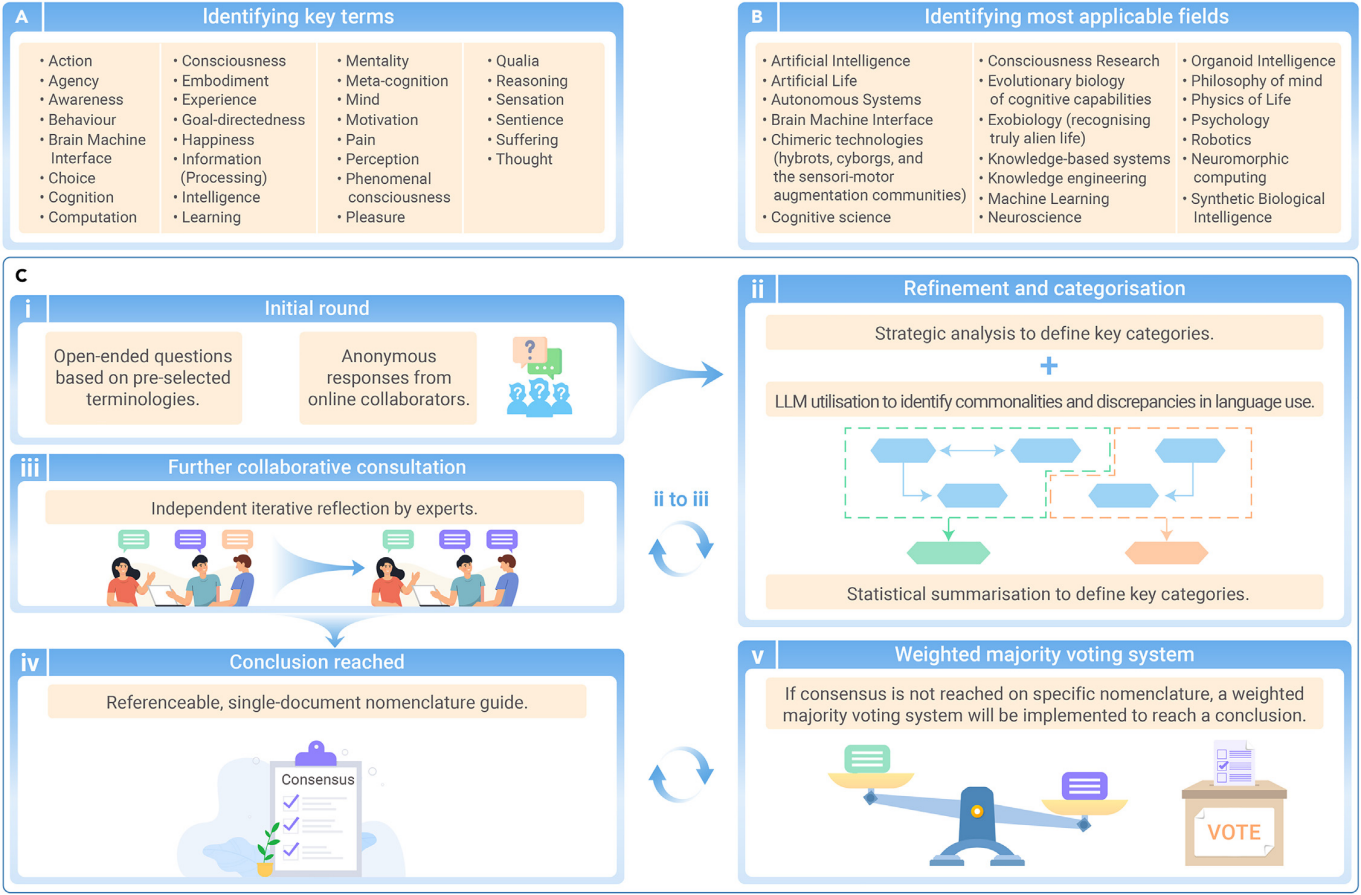
Initial key terms, most applicable fields, and core approach toward consensus (A) Proposed key terms to define. (B) Proposed most applicable specific fields the nomenclature guide will be used in; however, others may also find this work useful. (C) A mixed method approach with a modified Delphi method. This approach entails an initial round with pre-selected open-ended questions (i), strategic refinement and categorization (ii), and collaborative consultation (iii) in an iterative manner (ii and iii) until a suitable level of consensus is achieved (iv). If consensus is not reached on any specific terms, a weighted majority voting system will be implemented to reach a conclusion (v).

Rapidly growing fields aiming to create generally intelligent systems are controversial with disagreements, confusion, and ambiguity pervading discussions around the semantics used to describe this myriad of technologies. For example, even 15 years ago, at least 71 distinct definitions of “intelligence” had been identified.^1^ The diverse technologies and disciplines that contribute toward the shared goal of creating generally intelligent systems further amplify disparate definitions used for any given concept.^2^ It becomes increasingly impractical for researchers to explicitly re-define every term that could be considered ambiguous in each paper. As such, key terminology (Figure 1A) has often been imprecise, with signifiers used interchangeably to represent different concepts that are seldom formally defined. Even if the use of glossaries were implemented in all future empirical literature to ensure internal consistency and explicit definitions, this would place an onerous burden on the readers, especially non-experts.

Moreover, it is essential to enable collaboration between these different fields, such as those described in Figure 1B. A common language is needed to recognize, predict, manipulate, and build cognitive (or pseudo-cognitive) systems in unconventional embodiments that do not share straightforward aspects of structure or origin story with conventional natural species.^3^ Previous work proposing nomenclature guidelines are generally highly field specific and developed by selected experts, with little opportunity for broader community engagement. The authority of these experts is often a matter of contention that we argue would be addressed by engaging the community the nomenclature guidelines aim to assist in the generation process.

For this reason, we invite researchers and scientists in related areas to collaborate, broadly agree upon, and adopt nomenclature for this field as an imperative. This work aims to provide utility and nuance to the discussion and offers authors an option to use language explicitly, unambiguously, and consistently, insofar as rapidly emerging fields will allow, through the adoption of nomenclature adhering to a theory-agnostic standard.

Here, we propose a pathway toward a nomenclature consensus that could be useful for fields seeking to develop intelligent systems and welcome multidisciplinary collaboration input from all relevant stakeholders.

## Identifying key terms

Here, we propose a non-exhaustive list of key terms this work aims to define (Figure 1A). The most challenging words to define are likely those related to complex processes or internal states that are attributed various degrees of moral significance. Such terms often trigger intuitive emotional responses from readers and must be handled sensitively. As such, the non-exhaustive list in Figure 1A are terms that, historically, have at least sometimes been signifiers for these complex processes or internal states.

Specifically, we propose theory-agnostic definitions that can be viewed as semantically distinct or synonymous. Part of our proposal is to work to provide said distinction between semantic independence and synonymity, enabling the maximization of nuance and utility of term usage, creating opportunities for more deliberate scientific discourse. It is also acknowledged that some terms are components of other terms. For example, “perception” may be considered a component of “consciousness” or may otherwise be considered distinct. Clarifying semantic usage of these terms is a key goal of the proposed work.

As such, we suggest that reference to the terminology be made with respect to two usages: terms that are used in a manner that is empirical and terms that are used in a nominally qualitative manner. Here, empirical terms relate to those phenomena that are measurable. In contrast, terms with nominally qualitative usage are aimed to be considered as representing subjective perspectives. In practice, this contrast should create distinctions between the use of language in the context of describing phenomena in an empirical sense, compared to more subjective but still important usages of the terms in everyday language. Distinctions in term usage are intended to avoid neglect of terminologies that are classified as empirical in consideration of the critique of terms that are typically used without an empirical meaning. By allowing authors of future work to simply flag their intended usage, this alone will simplify communicating an important nuance. Where some words can be used in either category, this should be specified to allow the intended meaning and context to be easily understood, as done in recent work.^4^ For example, “phenomenal consciousness” is usually a nominally qualitative term, as no robust measures have been developed to provide empirical evidence of this phenomenon, and even robust definitions are challenging. Therein, the term phenomenal consciousness cannot—using current measures—be subjected to an empirical meaning. In contrast, “learning” can be measured empirically through changes in observable behavior and is also likely quantifiable through other means. While it is recognized that learning is a cognitive process that is unable to be conclusively observed with current methods, its operationalization across time does allow for empirical measurement. Moreover, as an example in biological organisms, known biophysical changes have been observed to be routinely associated with learning. Comparatively, while phenomenal consciousness can potentially be operationalized by developing quantitative metrics to capture this under an empirical framework, its real-life observation remains undetermined and uncertain at the current time, thus limiting the opportunity for its subjugation to an empirical conclusion. Furthermore, it is the unempirical nature of nominally qualitative terms that engenders avoidance of constraints on their understanding through attribution of a local minimum of our understanding of such phenomena. At the very least, should an author wish to approach the matter of a term usually positioned in a nominally qualitative manner with an empirical perspective, having a standard positioning will simplify the communication of this attempt. Having said this, we recognize that without firm definitions, even the above examples remain a subject for disagreement, a fact that only serves to highlight the importance of a consensus-based approach to seeking shared definitions of these terms. Identifying usages applicable to both categorizations would be a key focus in the proposed nomenclature guidelines.

## Pathway toward consensus

This work is intended to be as broadly collaborative as possible to ensure that it captures the diversity and breadth of the various researchers, philosophers, psychologists, bioethicists, sociologists, historians, scientists, etc., who may wish to use the nomenclature. However, as the very nature of diversity inherently means that this will attract many differences of opinion, it is vital that well-established methods for consensus-making are established to ensure that eventual conclusions can be reached. Multiple methods of consensus-making have been proposed and actively investigated in recent years.^5^^,^^6^^,^^7^^,^^8^^,^^9^ The development of these guidelines will also naturally leverage insights from linguistics via the influence that existing definitions have on the individual understanding of these terms and concepts.

Here, we propose following a mixed method approach with a modified Delphi method as the primary foundation. This method involves an initial round where fairly open-ended questions are used to solicit expert opinions, followed by subsequent rounds of refinement until a suitable level of consensus is achieved by applying the three key characteristics of the Delphi method.^10^ These are controlled feedback from participants, anonymity, and statistically summarized group responses. Independent reflection by experts in an iterative fashion will facilitate the modification of opinion relating to items in consideration of other responses, enabling equal opportunity in contribution to idea formation and, more importantly, collaboration between experts in areas of interest. By including preselected terminologies, it is also proposed to improve initial round response rates and ensure that initial terminologies utilized are grounded in an appropriate scholarly context. Moreover, the asynchronous and online format of this process will enable efficient data gathering and collaboration regardless of geographical location, attenuating participant attrition. In addition, this approach provides participants with initial anonymity—enabling a non-adversarial environment and reduction in bias, which may be encountered in face-to-face formal consensus methods. Should this method fail, other consensus-making methods will be applied to ensure optimal collaborative outcomes.^5^^,^^6^^,^^7^^,^^8^^,^^9^

To implement this process, a targeted survey questionnaire will be shared with all collaborators (Figure 1Ci). To provide a starting point unbiased by any single human perspective, we propose using large language models (LLMs) such as GPT-4-Turbo or open-source language models. LLMs, with their advanced natural language processing capabilities trained on a huge corpus of literature and other documents, can efficiently analyze existing definitions related to intelligent systems. By processing a vast array of academic papers, discussions, and existing nomenclature, these models can identify commonalities and discrepancies in the usage of the key terms flagged above. This synthesis of information can effectively and efficiently establish a baseline for discussions among multidisciplinary stakeholders. Furthermore, LLMs can assist in drafting and revising documents during the consensus-building process, ensuring clarity and coherence in the presentation of ideas and terminologies. This helps in creating a comprehensive and well-organized repository of terms as a starting point for the modified Delphi method.

Once the initial survey is completed and responses are gathered, qualitative methods will be used to refine these answers into key categories of answers where sufficient overlap exists. Additional consultation with all collaborators will be repeated until a consensus is ideally reached (Figures 1Cii-iv). As the multidisciplinary nature of this collaborative work may itself be a cause for misunderstanding, LLMs can further serve as an intermediary tool that translates complex concepts across different disciplines, making them more accessible and understandable to all involved. LLMs can rephrase and contextualize these viewpoints, facilitating a more productive and less ambiguous dialogue (Figure 1Cii). Additionally, these models can generate summaries and comparisons of different perspectives, helping to pinpoint areas of agreement and contention. This is particularly useful in managing and summarizing feedback from wider community consultations, ensuring that all voices are heard and considered in the nomenclature consensus. In the case that consensus is not reached on any specific terms (Figure 1Civ), a weighted majority voting system will be implemented to reach conclusions (Figure 1Cv). Although it is acknowledged that this will likely result in some terms that do not have full concordance from all collaborators, it is hoped that with a good-faith approach and fair consensus-making methods, coupled with a focus on nuance and utility, the result will be a nomenclature guide that is ultimately more useful than the current state of language usage.

## Identifying most applicable fields

This work should yield a nomenclature guide that is applicable to the fields described in Figure 1B and potentially more that are not currently listed. The eventual outcome of this work is a useful field guide for researchers exploring an intersection of these areas who are engaged in the development of diverse generally intelligent systems. If members of these fields or others find the nomenclature guidelines useful, it is hoped that the utility of referencing a single document will ease scientific communication and promote clarity for future work.

We invite all interested collaborators to register their interest at https://corticallabs.com/nomenclature.html to take part in this collaborative endeavor.

## References

[1] Legg S., Hutter M. (2007). Frontiers in Artificial Intelligence and Applications.

[2] Kagan B.J., Gyngell C., Lysaght T. (2023). The technology, opportunities, and challenges of Synthetic Biological Intelligence. Biotechnol. Adv..

[3] Fields C., Levin M. (2022). Competency in navigating arbitrary spaces as an invariant for analyzing cognition in diverse embodiments. Entropy.

[4] Smirnova L., Caffo B.S., Gracias D.H. (2023). Organoid intelligence (OI): The new frontier in biocomputing and intelligence-in-a-dish. Front. Sci..

[5] Zhao S., Lei T., Liang C. (2022). A consensus-reaching method for large-scale group decision-making based on integrated trust–opinion similarity relationships. Comput. Ind. Eng..

[6] Qin J., Ma X., Liang Y. (2023). Building a consensus for the best-worst method in group decision-making with an optimal allocation of information granularity. Inf. Sci..

[7] Gong G., Li K., Zha Q. (2023). A maximum fairness consensus model with limited cost in group decision making. Comput. Ind. Eng..

[8] Liang X., Guo J., Liu P. (2022). A large-scale group decision-making model with no consensus threshold based on social network analysis. Inf. Sci..

[9] Ren P., Wang X., Xu Z. (2022). Hesitant fuzzy linguistic iterative method for consistency and consensus-driven group decision making. Comput. Ind. Eng..

[10] Olsen A.A., Wolcott M.D., Haines S.T. (2021). How to use the Delphi method to aid in decision making and build consensus in pharmacy education. Curr. Pharm. Teach. Learn..

